# Psychosocial Support Promoting Recovery Among Women Survivors of Domestic Violence After Separation in China: A Qualitative Study

**DOI:** 10.3390/bs16060884

**Published:** 2026-06-01

**Authors:** Yingying Cui, Nur Saadah Mohamad Aun, Mohd Suhaimi Mohamad, Surendran Rajaratnam

**Affiliations:** Programme of Social Work, Centre for Research in Psychology and Human Well-Being, Faculty of Social Sciences and Humanities, Universiti Kebangsaan Malaysia, Bangi 43600, Malaysiamsuhaimi@ukm.edu.my (M.S.M.); surendran@ukm.edu.my (S.R.)

**Keywords:** domestic violence, Chinese women, recovery, psychosocial support, qualitative study

## Abstract

Domestic violence remains a critical social issue with long-term impacts on women’s well-being. Even after separation, women survivors still face multiple challenges in rebuilding their lives. Psychosocial support has been increasingly recognized as a key factor in facilitating recovery. This study employed a qualitative design to conduct semi-structured in-depth interviews with ten women survivors of domestic violence in China. Data were analyzed using thematic analysis to explore how psychosocial support promotes their recovery after separation. The findings identified four interrelated forms of psychosocial support: emotional support, informational support, tangible support, and companionship support. Each type of support contributed to recovery through distinct but interconnected pathways, including emotional stabilization, enhanced decision-making capacity, reduction in material stress, and reconstruction of social connectedness. These forms of support were derived from both informal networks and formal systems. While formal support services were available, survivors mainly relied on informal support networks, with limited access to and utilization of formal resources. These findings highlight the need for more accessible, culturally responsive, and integrated psychosocial support systems. Strengthening formal services while recognizing the role of informal support networks is essential for promoting survivors’ long-term recovery and well-being.

## 1. Introduction

Domestic violence remains one of the most pervasive forms of gender-based violence worldwide, with enduring impacts on women’s health, safety, and social well-being (Narayanan et al., 2024; White et al., 2024). According to data published by the World Health Organization in 2021, approximately one in three women globally has experienced physical or sexual violence by an intimate partner during their lifetime (WHO, 2021). Domestic violence not only causes immediate physical injuries but also results in long-term psychological consequences and broader socioeconomic difficulties (Khaironisak et al., 2017; Masih et al., 2024). Although leaving an abusive relationship may reduce immediate exposure to violence, the post-separation period often presents new challenges for survivors (Sontate et al., 2021; Spearman et al., 2024). Research has shown that many women survivors encounter significant barriers when attempting to rebuild their lives after leaving abusive partners (Carman & Kay-Lambkin, 2022; Said & Kaka, 2023). These challenges may include emotional trauma, economic hardship, limited employment opportunities, and childcare responsibilities resulting from prolonged abuse (Chua et al., 2023; Goodman-Williams et al., 2023). Recovery from domestic violence is a complex and long-term process that involves emotional healing, social reintegration, and the reconstruction of independent lives (Kavanagh & Fassbender, 2024). 

A growing body of research highlights the importance of psychosocial support in facilitating survivors’ recovery from domestic violence (Schucan Bird et al., 2025). Psychosocial support encompasses a range of supportive resources that address both psychological and social needs, including emotional encouragement, informational guidance, practical assistance, and opportunities for social interaction (Y. Wang et al., 2021). Emotional support from family members and friends can help survivors cope with trauma and restore self-confidence (Almış et al., 2020). Informational support such as legal advice, employment information, and service referrals helps survivors understand available resources and make informed decisions about their future (Schmidt et al., 2023; Hu et al., 2022). Tangible support, including financial assistance, housing support, and medical services, addresses survivors’ immediate material needs and reduces stress during the transition period (Goodman-Williams et al., 2023). In addition, companionship support through peer networks and community participation helps survivors rebuild social relationships and overcome feelings of isolation (R. L. Davies et al., 2025). These forms of psychosocial support play a critical role in helping survivors cope with post-separation challenges and regain a sense of stability and well-being (M. Davies et al., 2026).

In the Chinese context, domestic violence has gradually received greater social and policy attention in recent years (Su et al., 2022). The enactment of the Anti-Domestic Violence Law in 2016 marked a significant milestone in China’s efforts to address domestic violence and provide legal protection for victims (Gu et al., 2022). Despite these developments, women survivors in China still face multiple challenges when seeking support and rebuilding their lives after separation (Yuan et al., 2024). Cultural norms emphasizing family harmony and marital stability may discourage women from disclosing abuse or seeking help outside the family (Chen, 2024). Divorce and family conflict may also carry social stigma in some communities, which can further limit survivors’ access to support resources (Zhang et al., 2025). In addition, formal support services such as shelters, counseling services, and legal aid programs remain unevenly distributed across regions, creating additional barriers for survivors attempting to access professional assistance (Hu et al., 2022).

Previous studies on domestic violence in China have largely focused on the prevalence of violence, risk factors, and barriers to help-seeking among survivors (Hu et al., 2021). While these studies have provided valuable insights into the structural and cultural constraints faced by survivors, relatively fewer studies have examined the recovery process after survivors leave abusive relationships (Carman & Kay-Lambkin, 2022). Moreover, existing research often emphasizes survivors’ experiences of victimization rather than exploring the supportive resources that contribute to their recovery and resilience (H. Li et al., 2024). As a result, there remains limited empirical understanding of how different forms of psychosocial support facilitate survivors’ recovery after separation in the Chinese sociocultural context. Another limitation of existing research is that different forms of support are often examined in isolation rather than as interconnected resources that jointly contribute to recovery (Seff et al., 2024). Survivors’ recovery processes are multidimensional, involving emotional healing, access to practical resources, rebuilding social networks, and restoring personal autonomy (M. Davies et al., 2026). Understanding how various forms of psychosocial support interact and complement each other is therefore essential for developing effective support systems for survivors. Qualitative research that explores survivors’ lived experiences can provide deeper insights into how these support mechanisms function in everyday life.

This study is guided by the psychosocial recovery model (IASC, 2007; WHO, 2013), which conceptualizes recovery from violence as a holistic process involving psychological healing, social support, and the rebuilding of meaningful social roles. The psychosocial recovery perspective emphasizes that survivors’ well-being is shaped not only by individual coping capacities but also by the availability of supportive relationships and community resources. According to this framework, recovery involves multiple interconnected dimensions, including emotional stabilization, restoration of social connections, access to practical resources, and the reconstruction of personal identity and autonomy. Psychosocial support therefore plays a central role in facilitating survivors’ recovery by helping them process traumatic experiences, regain confidence, and reconnect with supportive social networks (Schucan Bird et al., 2025). Therefore, this study aims to provide a deeper understanding of how the different forms of psychosocial support assist survivors in coping with post-separation challenges.

## 2. Materials and Methods

### 2.1. Research Design 

This study adopted qualitative research using a case study approach and semi-structured in-depth interviews to generate rich, contextualized, and detailed information. Qualitative research is particularly appropriate for this study, which seeks to explore complex social phenomena and address “how” and “why” questions related to lived experiences, meanings, and processes (Denzin & Lincoln, 2011). This study aims to explore how psychosocial support contributes to the recovery of women survivors of domestic violence after separation in China. Through interactions with the research participants, a case study approach enables researchers to generate rich, contextually embedded insights into participants’ experiences and to identify patterns across individual narratives (Tisdell et al., 2025). Furthermore, semi-structured in-depth interviews are widely recognized as suitable for research involving sensitive topics that domestic violence may involve, as they create a private and empathetic space for participants to share personal experiences that may not emerge in structured or group settings (Liamputtong, 2006).

### 2.2. Sampling Strategy 

This study used purposive sampling to recruit women survivors of domestic violence; it enables the selection of participants based on the needs of research objectives and criteria (Patton, 2014). China’s 2016 Anti-Domestic Violence Law defines domestic violence as physical, psychological, or other forms of harm inflicted between family members through actions such as beatings, restraints, injury, restrictions on personal freedom, and repeated verbal abuse or intimidation. In this study, domestic violence refers to physical, emotional and sexually violent behaviors committed by husbands against their wives. The concept of a “survivor” of domestic violence is grounded in feminist scholarship and advocacy discourse, emphasizing agency, resilience, and the active process of reclaiming autonomy rather than victimhood (Brison, 2023; Cattaneo et al., 2021). In this study, survivors refer to these women who have experienced domestic violence and finally through divorce left the abusive relationship. According to the purpose of the research, those participants who could provide rich and relevant information regarding psychosocial support and recovery after separation from abusive relationships have been selected. The focus on the post-separation stage aligns with research suggesting that recovery and support needs often intensify after leaving the abusive relationship (Ford-Gilboe et al., 2015). Those who have only recently left an abusive relationship may struggle to identify which factors or supports are beneficial or detrimental (Arendt, 2022). Therefore, this study selected women survivors who have received services at Jinan Anti-Domestic Violence Shelter (JADVS) of Shandong Province and left the abusive relationship through divorce more than one year. As all participants were recruited through a shelter-based support context, they have greater exposure to formal support than the broader population of survivors. A total of ten women survivors participated in the study. Recruiting participants was particularly challenging in the Chinese context due to the sensitive nature of the topic, concerns about stigma and privacy, fear of re-traumatization, and survivors’ reluctance to disclose personal experiences of abuse (Yuan et al., 2024). These factors may reduce survivors’ willingness to participate in domestic violence research and limit access to potential participants. Nevertheless, the sample size aligns with qualitative research standards emphasizing depth over breadth, where smaller samples can yield detailed and meaningful insights when participants share relevant experiences (Guest et al., 2006). Data saturation was considered achieved when no substantially new themes emerged from subsequent interviews, indicating adequate coverage of the phenomenon (Braun & Clarke, 2021). Initial thematic repetition became evident after the eighth interview, and two additional interviews were conducted to confirm the consistency and adequacy of the identified themes.

### 2.3. Data Collection

After approval by the head of JADVS, the participants were contacted and subsequently invited to sign the informed consent form. Individual interviews were mainly conducted through WeChat voice calls according to the participants’ preferences. Each interview lasted from 45 min to 1 h to ensure meaningful and productive conversation. Eight participants were interviewed once. Two were interviewed twice because of health-related issues. Participants were asked to confirm that they were in a private and safe space and were free to speak without interruption. Participants were encouraged to use earphones and were informed that they have the right to withdraw at any time. The researchers used digital voice-recorded devices for audio recording upon receiving consent from all the participants. The information upon collection was kept confidential, and only the researchers had access to the data which were stored on the researchers’ computer.

The interview protocol included: (i) experiences of domestic violence and the decision to divorce, (ii) what kind of challenges have been encountered during the post-separation period, (iii) what kind of psychosocial support have been received, and (iv) perceptions of how these forms of support influenced recovery. Initially, three survivors were invited to participate in pilot interviews to assess the clarity, sensitivity, and sequencing of questions. Based on feedback from these preliminary interviews, several questions were rephrased to ensure clarity and reduce potential emotional distress. During the main interviews, careful attention was paid to the wording, order, and tone of questions. An open and flexible interviewing approach was maintained to allow participants to guide the depth and direction of their narratives. Rather than adhering rigidly to the interview guide, the interviewer adapted follow-up questions based on participants’ responses, encouraging elaboration and clarification where necessary. Throughout the interview process, efforts were made to minimize interviewer bias.

### 2.4. Data Analysis

The data analysis of this study followed the six-phase process outlined by Braun and Clarke (2013): (1) we familiarized ourselves with the data, transcribed the data, repeatedly read through it, and recorded initial ideas. (2) We generated initial codes; the coding process was primarily manual, and initial codes were derived inductively from the data. Similar codes were then grouped into broader categories. (3) We searched for themes, grouped codes into potential themes and gathered all data relevant to each potential theme, then identified the potential themes and sub-themes. (4) We reviewed themes; this involved reviewing whether the themes align with the coded data and the overall dataset. If the data did not adequately support a theme or showed too much variation, the theme potentially needed to be renamed or reclassified as a sub-theme. (5) We defined and named themes and generated clear definitions and names for each theme to ensure consistency in meaning across all themes. (6) We produced the report, analyzed the selected vivid and compelling extracts, linked the findings to the research questions and the existing literature, and presented the results in a coherent and scholarly report.

### 2.5. Trustworthiness

In qualitative research, trustworthiness is considered the most appropriate criterion for evaluating research quality, replacing the positivist notions of validity and reliability (Lincoln, 1985). To ensure credibility, dependability, confirmability, and transferability, several strategies were integrated throughout the research process. Credibility was enhanced through purposive sampling and prolonged engagement with participants, which facilitated trust-building and encouraged open sharing of experiences (Patton, 2014). Member checking was conducted during interviews by summarizing and clarifying participants’ responses to ensure the accuracy of interpretation (Creswell, 2014). Dependability was strengthened through a systematic analytic process using reflexive thematic analysis, supported by an audit trail documenting coding procedures, analytic decisions, and reflective memos (Braun & Clarke, 2021). Confirmability was addressed through reflexive practices, including maintaining a research journal to monitor assumptions and minimize researcher bias (Friesen et al., 2012). Finally, transferability was supported through rich, thick descriptions of participants’ experiences and the sociocultural context of post-separation recovery in China, allowing readers to assess the applicability of findings to other settings (Shenton, 2004). Together, these strategies enhanced the rigor and transparency of the study and ensured that the findings authentically represented the lived experiences of women survivors of domestic violence after separation.

### 2.6. Reflexivity

The researcher remained aware that personal assumptions, cultural background, and prior understanding of domestic violence could influence data collection and interpretation. Throughout the research process, reflexive practices such as memo writing and continuous self-reflection were used to enhance awareness of potential biases and maintain sensitivity to participants’ experiences. Particular attention was paid to creating a supportive and non-judgmental interview environment when discussing sensitive experiences related to domestic violence (Denzin & Lincoln, 1994).

## 3. Results

### 3.1. Participants

This study recruited a total of 10 women survivors of domestic violence. Table 1 shows the detailed demographic information of the participants.

### 3.2. Themes

**Figure 1:**
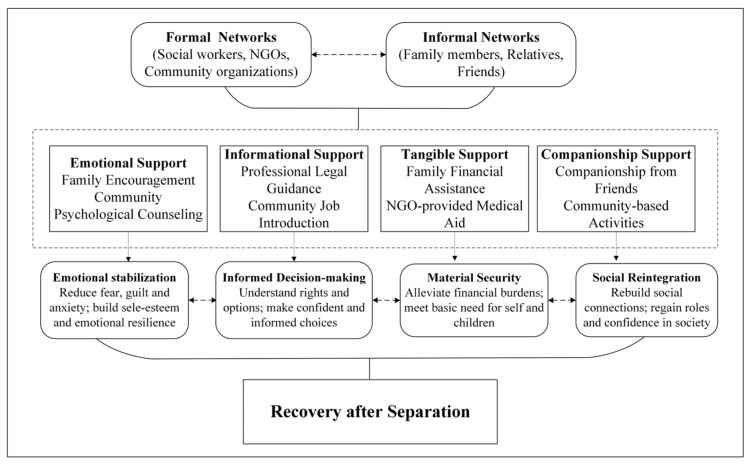
Thematic map of psychosocial support among survivors after separation. Note: Solid arrows indicate directional contributions, dashed double-headed arrows represent interrelated and reciprocal relationships.

The analysis of interview data identified four major forms of psychosocial support experienced by women survivors of domestic violence after separation: emotional support, informational support, tangible support, and companionship support. These forms of support were provided through both formal and informal networks, including family members, relatives, friends, social workers, NGOs and community organizations (Figure 1).

#### 3.2.1. Emotional Support

**Family Encouragement.** Family encouragement mainly came from parents, children, siblings and other relatives. Some survivors (*n = 5*) explained that supportive attitudes from their parents helped them feel accepted and understood. Survivor 3 said: “*My mother always told me that I should take care of myself first and not blame myself for what happened. Talking with her made me feel calmer.*” The children were also described as an important source of emotional motivation. For all the participants, the responsibility of protecting and caring for their children strengthened their determination to move forward and rebuild their lives (*n = 10*). Survivor 5 said: “*When I thought about my children, I knew I had to stay strong. I wanted to create a safer life for them.*” Survivors who received care and support from siblings and other relatives described feeling less isolated and more confident about facing future challenges (*n = 3*).

*After my ex-husband put me in the hospital, my parents, brother, and sister-in-law were by my side, taking care of me. They held my hand and cried, saying, “You have to stay strong, fight, and live on.*

(Source: Survivor 2)

**Community Psychological Counseling.** In addition to family support, some survivors accessed emotional assistance through community psychological counseling. The counseling service encourages survivors to openly express their emotions and discuss their abused experiences without fear of judgment (Lakin et al., 2022). Two survivors explained that professional counseling helped them process the emotional trauma caused by prolonged violence. Survivor 4 said: “*After the divorce, I was in a really bad emotional state and often had negative thoughts…so I went to the community center to seek psychological counseling, this has helped me a lot.*” This experience was particularly meaningful for survivors who had previously lacked opportunities to talk about their experiences. Survivor 1 said: “*Before I spoke with the counselor, I kept everything inside. During the sessions I finally felt that someone truly listened to my story.*” Two survivors also reported that psychological counseling helped them better understand their emotional reactions after separation. This understanding reduced self-blame and allowed survivors to gradually regain emotional stability. Survivor 3 said: “*The counselor helped me realize that feelings such as fear, guilt, and anxiety were normal after experiencing violence. That made me feel less confused about my emotions.*” Furthermore, counseling services provided survivors with strategies to cope with emotional stress and rebuild confidence. Survivor 7 reported learning ways to manage negative emotions and developing a more positive outlook on their future lives.

*The community provided me with psychological counseling to help me work through my mental and emotional struggles. When I first found out I was paralyzed, I couldn’t accept the reality…But after receiving counseling, I gradually calmed down. Thinking about my parents and my child, I knew I had to stay strong and fight for justice.*

(Source: Survivor 7)

#### 3.2.2. Informational Support

**Professional Legal Guidance.** Professional legal guidance was mainly provided by legal aid institutions, and it can help survivors understand available legal options and navigate complex legal processes. Several survivors described that accurate legal information enabled them to better understand the steps required to secure their safety and protect their children (*n = 4*). Survivor 1 said: “*I applied for legal aid through the community. They guided me in preparing all the necessary documents for the custody change and helped me go through the related procedures.*” In addition, two survivors also noted that legal professionals often provided practical advice on collecting evidence, filing legal documents, and interacting with authorities. This information allowed survivors to approach legal processes with greater confidence and clarity. Survivor 9 said: “*The community staff helped me get in touch with a legal TV program…They guided me on how to prepare the litigation documents and how to follow up on the legal process.*”

**Community Job Introduction.** Information about employment opportunities provided by community organizations played a significant role in helping survivors regain financial independence. Several survivors described how community workers helped connect them with potential job opportunities or training programs (*n = 3*). These introductions often served as an important starting point for survivors seeking to re-enter the workforce. Survivor 4 said: *“After the divorce, I attended a job fair organized by the community and found a job as a babysitter. Although taking care of kids every day is exhausting, it allows me to support myself.*” In addition, some survivors highlighted that these opportunities not only brought financial relief but also helped them regain confidence after years of being economically controlled by their abusers (*n = 4*).

*I went to the community center and told them about my situation. The staff registered my information and helped me find a housekeeper job. I’m really very grateful to the community. The first day at work was a moment when I felt free again, earning money for myself, not under someone’s thumb’. This job has enabled me to achieve self-sufficiency.*

(Source: Survivor 5)

#### 3.2.3. Tangible Support

**Family Financial Assistance.** The financial support from parents or relatives provided an important safety net for survivors after separation. Several survivors reported that their parents or extended family members helped them cover basic living expenses such as housing, food, and childcare (*n = 8*). This assistance reduced immediate economic pressure and allowed survivors time to search for employment or adjust to new living arrangements. Survivor 6 said: “*After I left, I did not have a stable income. My parents helped me with rent and daily expenses for a while. Their support helped me get through the most difficult period.*” Two survivors also reported that their parents used personal savings or even borrowed money from relatives to support them. Survivor 2 said: “*My parents, brother, and sister-in-law have spent all their savings, and we’ve borrowed a lot from relatives. This has placed a heavy burden on my family.*” In addition, Survivor 8 described receiving substantial financial and caregiving support from parents and relatives during times of severe hardship.

*My parents have to look after me in all aspects of daily life. They took out the savings they had worked hard for over half their lives to pay for my surgery. Relatives also extended a helping hand, lending me money so I could have the surgery as soon as possible.*

(Source: Survivor 8)

**NGO-provided Medical Aid.** Many survivors experienced physical injuries or long-term health problems resulting from domestic violence. Two survivors reported receiving medical assistance from NGOs. Survivor 3 reported that NGOs helped her access medical examinations, treatment services, or referrals to healthcare institutions. She said: “*After the incident, I needed medical care, but I was worried about the cost. The organization helped me arrange treatment and guided me through the process.*” In addition, organizations also assisted survivors in obtaining affordable medical services or financial support for treatment. These resources were particularly valuable for survivors who faced financial constraints after separation.

*I shared my experience on social media, which drew widespread attention and support from netizens. I also received help from anti-domestic violence volunteers and related Non-Governmental Organizations. They assisted me in obtaining injury and disability assessments and helped me apply for medical assistance funds provided by the Yuanzhong Family and Community Development Service Center.*

(Source: Survivor 2)

#### 3.2.4. Companionship Support

**Companionship from Friends.** Friends often provided companionship through everyday activities such as conversations, shared meals, or simply spending time together. Two survivors described that spending time with trusted friends helped them relieve emotional stress and reminded them that they were not facing their difficulties alone. Survivor 1 said: “*Sometimes my friends would call me or invite me out to talk. Just chatting with them made me feel much better.*” In addition to providing emotional comfort, supportive friendships gradually helped survivors rebuild trust and regain a sense of normalcy. Survivor 5 said: “*My friends encouraged me to go out and do things together. Slowly, I started to feel that life could become normal again.*” Survivor 2 also noted that friends often encouraged her to re-engage with everyday life by inviting her to participate in social activities. This helped her regain confidence in social interactions and strengthened her motivation to move forward.

*After the divorce, I stayed at a few friends’ houses. They helped me get phone numbers and rent apartments using their IDs. At that time, I was afraid to go out alone and worried that my ex-husband would find me. My friends always accompanied me and took me out shopping and relaxing. I really appreciated my friends’ company during that time.*

(Source: Survivor 2)

**Community-based Activities.** Community-based activities provided survivors with opportunities to interact with others, share experiences, and gradually rebuild their social networks (Machisa et al., 2018). Two survivors explained that joining community programs or local activities helped them regain a sense of belonging. These activities allowed survivors to engage with others in supportive environments, reducing feelings of isolation after separation. Survivor 7 said: “*When I started joining community activities, I met people who were willing to talk and listen. It made me feel that I was part of the community again.*” In addition, community activities also created opportunities to share experiences with others facing similar challenges, which helped survivors feel understood and less alone in their recovery journeys. Survivor 5 said: “*When I joined the community activities, I realized that other women had gone through similar experiences. Listening to their stories made me feel that I was not the only one facing these difficulties.*” Furthermore, participation in community activities helped survivors shift their focus from past trauma toward future possibilities. Survivor 5 reported that these interactions gradually rebuild their confidence and strengthened their sense of social inclusion.

*I was invited to participate in a cooking competition organized by the community. Since I’m pretty good at cooking, I even won a prize. Even though I’ve lost my family, I found a sense of home within the community.*

(Source: Survivor 5)

## 4. Discussion

This study explored how psychosocial support contributes to the recovery of women survivors of domestic violence after separation in China. The findings reveal that survivors’ recovery is supported by multiple forms of psychosocial support, including emotional support, informational support, tangible support, and companionship support. These forms of support were provided through both informal networks such as family members and friends, and formal systems including community organizations, social workers and non-governmental organizations. Together, these supportive resources helped survivors cope with the emotional consequences of abuse, access necessary information and resources, stabilize their living conditions, and rebuild social connections. Guided by the psychosocial recovery model, how different forms of psychosocial support function as important recovery resources deserves further discussion.

Emotional support plays a critical role in coping with the psychological consequences of domestic violence (Goodman et al., 2016; Sullivan, 2018). The findings of this study indicated that encouragement from parents, children, and extended family members play an important role in helping survivors feel understood, valued, and supported after separation. This is consistent with prior research demonstrating that supportive family relationships can significantly reduce survivors’ feelings of isolation and self-blame, while enhancing their psychological resilience and emotional well-being (Anderson et al., 2012; Goodman-Williams et al., 2023). From a psychosocial recovery perspective, such family-based emotional support functions as a key protective factor by strengthening survivors’ sense of belonging and emotional security (Wessells & Kostelny, 2022). Emotional support is also an important mechanism through which survivors begin to reconstruct their emotional stability and interpersonal trust (Sapkota et al., 2022). At the same time, this study confirms the importance of community psychological counseling in supporting survivors’ emotional recovery. Counseling services provided survivors with safe and confidential environments where they could openly express their emotions and process traumatic experiences without fear of judgment. Previous research has similarly shown that trauma-informed counseling and psychosocial interventions can facilitate emotional regulation and reduce symptoms of anxiety, depression, and post-traumatic stress among survivors of violence (M. Davies et al., 2026; Lakin et al., 2022).

However, in the Chinese sociocultural context, emotional support is primarily derived from family members, while access to formal psychological services remains relatively limited. This pattern is consistent with prior research indicating that help-seeking among Chinese women survivors tends to rely heavily on informal support networks rather than professional services (Hu et al., 2021; Liang et al., 2018). This reliance on family support can be understood within the broader collectivist cultural framework, where interpersonal relationships, familial obligations, and the maintenance of social harmony are highly valued (Liu et al., 2026). At the same time, structural barriers such as the limited availability of specialized services, low awareness of psychological support, and concerns about stigma continue to restrict survivors’ engagement with formal support systems (Y. Wang et al., 2021; Zhang et al., 2025). As a result, although community counseling services are available, only a minority of survivors can access or utilize such resources in practice. This gap between the availability and accessibility of formal support highlights an important limitation in current support systems and underscores the need to improve the reach and cultural responsiveness of psychological services for survivors of domestic violence in China.

Informational support represents a critical dimension of psychosocial recovery by enabling survivors to better understand their rights, access resources, and make decisions. Consistent with the existing literature, access to relevant and timely informational support plays an essential role in enhancing survivors’ autonomy and facilitating their transition to independent living (Postmus et al., 2020; Sullivan, 2018). Legal information including knowledge of protection orders, divorce procedures, and child custody arrangements helped survivors navigate complex institutional systems and reduce uncertainty during the post-separation period. This finding aligns with prior research demonstrating that legal knowledge can empower survivors by increasing their perceived control and reducing vulnerability to further abuse (Cordier et al., 2021; Trabold et al., 2020). From a psychosocial recovery perspective, such informational support contributes to the development of self-efficacy and decision-making capacity, both of which are essential for long-term recovery (Ogbe et al., 2020). Similarly, employment-related information provided through community channels played a significant role in supporting survivors’ economic reintegration. Access to job opportunities not only improved survivors’ financial independence but also contributed to their sense of purpose and social participation. Previous studies have highlighted that economic empowerment is closely linked to survivors’ well-being and their ability to sustain separation from abusive partners (Johnson et al., 2022; Stylianou, 2018). In this study, informational support related to employment thus functioned not merely as practical assistance but as a key pathway toward rebuilding autonomy and restoring self-worth. 

The recent research indicates that community-based and institutional services in China have gradually expanded their roles in providing legal and employment-related support for vulnerable groups, including survivors of domestic violence (Cheng et al., 2025; X. Wang et al., 2020). However, only a small proportion of survivors reported accessing or benefiting from these formal services. This indicates a significant gap between the availability and actual utilization of formal informational support. In other words, the limitation does not necessarily lie in the absence of formal support, but rather in its restricted accessibility, limited reach, and uneven utilization among survivors. This pattern has been widely observed in recent studies, which highlight that the underutilization of formal support services is a persistent issue in domestic violence contexts, particularly in non-Western societies (Cheng et al., 2025; Childress et al., 2022; Goodson & Hayes, 2021). Several factors may account for this discrepancy. First, survivors may lack awareness of available legal and employment-related services, particularly when information dissemination is insufficient or not effectively targeted. Prior research has shown that limited knowledge about rights and services remains a major barrier to help-seeking among women survivors (Y. Li et al., 2024; Meyer, 2016). Second, institutional procedures may be perceived as complex, time-consuming, or difficult to navigate, which discourages engagement. This aligns with findings that bureaucratic barriers and fragmented service systems often reduce survivors’ willingness to engage with formal institutions (Cheng et al., 2025; Song et al., 2021). Third, sociocultural factors such as mistrust of formal institutions, concerns about stigma, or a preference for resolving issues within familiar social networks may further reduce survivors’ willingness to seek formal informational support (Hu et al., 2021; Liang et al., 2018; Zhang et al., 2025). In collectivist cultural contexts, relational trust and informal networks often take precedence over institutional channels, shaping help-seeking behaviors in significant ways (Sripada, 2021). Informational support should be understood not merely as the availability of resources, but as a process shaped by accessibility, awareness, and sociocultural acceptability (Postmus et al., 2020; Sullivan, 2018). Strengthening outreach, simplifying service procedures, and enhancing trust in formal systems may be essential to improving the effectiveness of informational support for survivors of domestic violence. Recent studies further suggest that integrating community-based outreach with culturally sensitive service delivery models can significantly improve survivors’ engagement with formal support systems (Green et al., 2024; Papas et al., 2023).

Tangible support constitutes a crucial dimension of psychosocial recovery by addressing survivors’ immediate material and health-related needs following separation. Adams et al. (2021) believe that financial assistance and access to medical care are fundamental in stabilizing survivors’ living conditions and reducing financial stress after separation. The findings of this study indicated that financial assistance emerged as a primary source of tangible support, enabling survivors to meet basic needs such as housing, medical expenses, and daily living costs. This is in line with prior research indicating that economic insecurity is a major barrier to leaving abusive relationships and that financial support from informal networks can play a critical role in facilitating survivors’ transition to independent living (Mellar et al., 2024; Postmus et al., 2020). From a psychosocial recovery perspective, such support not only alleviates immediate economic pressure but also contributes to survivors’ sense of stability and security, which are essential preconditions for psychological recovery (Williams et al., 2024). 

In addition, medical aid provided by NGOs represents a form of formal tangible support that complements family-based assistance. Such support plays a particularly important role for survivors experiencing physical injuries or long-term health consequences of abuse. Previous research has shown that access to medical and health-related services is closely associated with improved physical and mental health outcomes among survivors of domestic violence (García-Moreno et al., 2015; Lutgendorf, 2019). In this study, NGO-provided medical aid helped reduce treatment costs and increased access to necessary healthcare services, thereby supporting both physical recovery and overall well-being. However, the findings also indicate that access to formal tangible support remains limited and uneven. Only a small number of survivors reported receiving assistance from NGOs, suggesting a gap between the availability of services and their actual reach. Recent studies have similarly highlighted that, despite policy efforts to expand social and health services, disparities in resource distribution and service accessibility persist across regions in China (Qi et al., 2020; Tam et al., 2016). Barriers such as limited service coverage, lack of awareness, and administrative complexity may further restrict survivors’ ability to access formal tangible support (X. Wang et al., 2020; Xue et al., 2024).

Companionship support represents an important dimension of psychosocial recovery by fostering survivors’ sense of connectedness and facilitating their reintegration into social life after separation. In line with previous studies, social isolation is a common consequence of domestic violence, and rebuilding social connections is a critical step in the recovery process (Goodman et al., 2016; Sylaska & Edwards, 2014). In this study, companionship from friends helped survivors re-establish trust in interpersonal relationships and regain confidence in engaging with others. Unlike family relationships, which may involve obligations or expectations, friendships often offer more voluntary, flexible, and non-judgmental forms of interaction. This aligns with previous research suggesting that peer and friendship networks can provide unique forms of support characterized by mutual understanding, emotional reciprocity, and reduced pressure (Robinson et al., 2020; Seff et al., 2024). In addition, participation in community activities further extended survivors’ social networks and created opportunities for meaningful social engagement. The interactions with individuals who had experienced similar challenges enabled survivors to share experiences, exchange coping strategies, and feel understood. This finding is consistent with studies highlighting the value of peer support and group-based interventions in promoting recovery among survivors of violence (Lakin et al., 2022; Sullivan, 2018). From a psychosocial recovery perspective, such interactions contribute to the process of normalization, whereby survivors come to understand that their experiences are not isolated, thereby reducing self-blame and stigma. At the same time, community participation enhances survivors’ sense of belonging and social identity, which are key factors in rebuilding well-being and resilience.

However, access to companionship support is shaped by sociocultural and structural conditions. In the Chinese context, survivors’ social participation may be constrained by factors such as stigma, fear of disclosure, and limited availability of supportive community spaces. Previous research has shown that concerns about “losing face” and maintaining social harmony can discourage survivors from openly discussing their experiences or engaging in broader social networks (Hu et al., 2021; Thomson et al., 2026). As a result, some survivors may remain socially withdrawn or rely on a limited circle of trusted individuals, which restricts the potential benefits of broader companionship support. In addition, while community-based activities provide valuable opportunities for social connection, their accessibility and inclusiveness may vary across regions. Uneven distribution of community resources and differences in service quality may limit survivors’ participation, particularly in less developed areas (Liu et al., 2026; Sun et al., 2022). This suggests that companionship support is also affected by the gap between availability and accessibility. This also can help explain why companionship support was reported less frequently among participants in this study. Strengthening community-based programs, promoting peer support networks, and reducing stigma may therefore be essential for enhancing the effectiveness of companionship support in the Chinese context.

To sum up, psychosocial support plays a vital and multifaceted role in promoting recovery among women survivors of domestic violence after separation. The findings of this study indicate that emotional, informational, tangible, and companionship support contribute to recovery through interconnected pathways, including emotional stabilization, enhanced agency, reduced material strain, and the rebuilding of social connectedness. Consistent with the psychosocial recovery model, recovery is shaped by the dynamic interaction between individual capacities and external support systems (Sullivan, 2018). However, a key insight of this study is that, within the Chinese context, psychosocial support is characterized by a structural imbalance: while both formal and informal support systems exist, survivors mainly rely on informal networks such as family and close social ties, whereas formal support services remain underutilized despite their availability (Hu et al., 2021; Xue et al., 2024). This gap reflects not only issues of accessibility and awareness but also the influence of sociocultural norms and institutional constraints. Therefore, promoting survivors’ recovery requires strengthening the accessibility, responsiveness, and trustworthiness of formal support systems and integrating the existing strengths of informal support networks. When these support systems function in a more balanced and coordinated manner, the negative impacts of post-separation stressors can be more effectively mitigated, thereby enhancing survivors’ capacity for long-term recovery and adaptation.

The findings of this study contribute to the psychosocial recovery literature by highlighting how survivors’ recovery processes are shaped not only by individual coping capacities but also by the accessibility and sociocultural acceptability of support systems. The study extends the psychosocial recovery perspective by demonstrating that formal and informal psychosocial support operate through interconnected pathways in facilitating recovery after separation. In particular, the findings highlight the structural imbalance between formal and informal support systems within the Chinese context, where survivors continue to rely primarily on family and close social networks despite the availability of formal services. From a practical perspective, the findings underscore the importance of developing more accessible, culturally responsive, and community-based support services, while strengthening coordination between formal institutions and informal support networks. Future research may further examine survivors’ long-term recovery trajectories across different regions and sociocultural settings, as well as explore how formal support systems can be better adapted to survivors’ lived realities and help-seeking preferences.

## 5. Conclusions

This study employed a qualitative research method to explore how psychosocial support promotes recovery among women survivors of domestic violence after separation in China. Guided by the psychosocial recovery model, we have a clear understanding of how different forms of psychosocial support interact with survivors’ lived experiences to facilitate recovery. These forms of support operate through multiple psychosocial pathways, including emotional stabilization, improved decision-making capacity, reduction in material stress, and reconstruction of social connection. Within the Chinese sociocultural context, support is embedded in relational networks and influenced by cultural values that emphasize family responsibility and interpersonal interdependence. As a result, informal support from family members, relatives, and friends plays a dominant role in survivors’ recovery, while formal support systems such as community services, legal assistance, and non-governmental organizations serve a complementary but limited function. This highlights a structural imbalance in the support system, where the availability of formal services does not necessarily translate into effective accessibility or utilization.

Service providers and social workers should adopt a holistic and survivor-centered approach to support women recovering from domestic violence after separation. They should help survivors identify their needs, access available resources, and strengthen their psychosocial capacities. In addition, it is important to strengthen formal support systems and improve their accessibility. Community-based services, legal aid, and employment support programs should be better integrated and more proactively delivered to survivors. Outreach strategies, simplified service procedures, and culturally sensitive interventions are needed to bridge the gap between service availability and actual utilization. Developing both online and offline support platforms, such as peer support groups and community-based programs, can further enhance mutual support among survivors and reduce feelings of isolation. Given the central role of family in the Chinese context, interventions should also incorporate family-oriented approaches. Providing guidance and support to family members can help reduce the emotional and financial burden placed on informal support networks. Strengthening collaboration between formal services and informal networks may contribute to a more balanced and sustainable support system. Finally, promoting a recovery-oriented perspective is essential. Encouraging survivors to recognize their strengths, learn from shared experiences, and gradually re-engage with social life can help them rebuild a sense of control and meaning. Such approaches are crucial for facilitating long-term recovery, enhancing well-being, and supporting survivors in rebuilding their lives with dignity and confidence.

## 6. Research Limitations

This study has several limitations. First, the relatively small sample was drawn from a single province in China, which may not fully capture the diversity of psychosocial support experiences among women survivors of domestic violence in other regions. In addition, regional disparities in economic development and the availability of social and institutional resources may influence the forms and accessibility of support, and thus the findings may not be fully representative of contexts beyond the study setting. Then, the data were based on participants’ retrospective accounts of their post-separation experiences. As such, their recollections may be subject to partial distortion or selective memory. However, it is important to note that qualitative research is concerned not only with objective events but also with how individuals interpret and make meaning of their experiences. Understanding survivors’ subjective perceptions of support and recovery is particularly important in the context of domestic violence, where emotional and psychological processes are central to the recovery trajectory. From an interpretivist perspective, reality is understood as socially constructed rather than objectively fixed, and individuals’ subjective experiences constitute a valid and meaningful form of knowledge (Winkler, 2005). Therefore, while recall bias cannot be eliminated, participants’ narratives provide valuable insights into how psychosocial support is perceived, experienced, and integrated into their recovery processes.

## Figures and Tables

**Table 1:** Demographic information of women survivors.

Characteristics	M (SD) or N (%)
**Age**	
Mean (SD)	39.6 (5.4)
**Education level**	
Junior high school	2 (20)
Senior high school	4 (40)
Junior college	4 (40)
**Occupation**	
Employed	7 (70)
Unemployed	2 (20)
Self-employed	1 (10)
**Number of children**	
One child	9 (90)
Two children	1 (10)
**Duration of marriage**	
1–10 years	4 (40)
10 years and above	6 (60)
**Duration of divorce**	
1–2 years	7 (70)
3–5 years	3 (30)
**Type of abuse experienced**	
Physical and emotional	8 (80)
Physical, emotional and sexual	2 (20)

## Data Availability

The datasets presented in this study are available on request from the corresponding author. The data is not publicly available to retain participant privacy.

## Abbreviations

The following abbreviations are used in this manuscript:

WHO	World Health Organization
NGO	Non-government organization
JADVS	Jinan Anti-Domestic Violence Shelter
IASC	Inter-Agency Standing Committee
